# Porokeratosis of Mibelli Treated With Topical 2% Lovastatin/2% Cholesterol Ointment

**DOI:** 10.7759/cureus.65871

**Published:** 2024-07-31

**Authors:** Julia Woźna, Katarzyna Korecka, Monika Bowszyc-Dmochowska, Magdalena Jałowska

**Affiliations:** 1 Department of Dermatology, Poznan University of Medical Sciences, Poznan, POL; 2 Department of Dermatology and Venereology, Poznan University of Medical Sciences, Poznan, POL; 3 Department of Dermatology, Cutaneous Histopathology and Immunopathology Section, Poznan University of Medical Sciences, Poznan, POL

**Keywords:** ultraviolet-induced fluorescence dermatoscopy, lovastatin/cholesterol ointment, isoprenoid pathway, porokeratosis of mibelli, porokeratosis

## Abstract

Porokeratosis is characterized by disruptions in the isoprenoid pathway, leading to the development of cornoid lamella, a unique skin lesion consisting of parakeratotic cells. The condition has a genetic foundation involving mutations affecting cholesterol synthesis, and new treatments aim to address these metabolic disruptions. This study examines a 56-year-old male with porokeratosis of Mibelli (PM) who presented with a non-healing erosion on his finger that persisted for two years. Previous therapies, including corticosteroids, antibiotics, and tacrolimus, proved ineffective. The patient then received a novel treatment with a topical 2% lovastatin/2% cholesterol ointment. After nine months, there was significant clinical improvement; the lesion was markedly reduced in size and appearance. This case underscores the potential of lovastatin/cholesterol ointment as an effective treatment for PM, indicating its promise for broader therapeutic applications.

## Introduction

Porokeratosis represents a diverse group of keratinization disorders originating from defects in the isoprenoid pathway. These entities are characterized microscopically by a distinct feature known as the cornoid lamella, which histopathologically corresponds to a vertical column of parakeratosis located over dyskeratotic cells within the granular layer [1].

The various forms of porokeratosis include disseminated superficial actinic porokeratosis (DSAP), porokeratosis of Mibelli (PM), disseminated superficial porokeratosis, palmoplantar porokeratosis, linear porokeratosis (LP), and verrucous porokeratosis. Other, less common clinical variants also exist, such as follicular porokeratosis, porokeratoma, and porokeratotic eccrine ostial and dermal duct nevus [1].

The data on treatment methods in these entities are scarce, and many different therapeutic approaches have been reported with very frequent unsuccessful outcomes. Similar to other clonal keratinocyte conditions, the treatment approaches for porokeratosis involve cryotherapy, photodynamic therapy, CO2 lasers, and 5-fluorouracil, acitretin, topical corticosteroids, and vitamin D analogs [1].

Recent advances in the understanding of the mevalonate pathway have led to the exploration of new methods, such as the use of topical statins with cholesterol [2]. Here, we present a patient with a PM who presented to the dermatology clinic with finger erosion and was successfully treated with topical 2% lovastatin/2% cholesterol ointment.

## Case presentation

**Figure 1:**
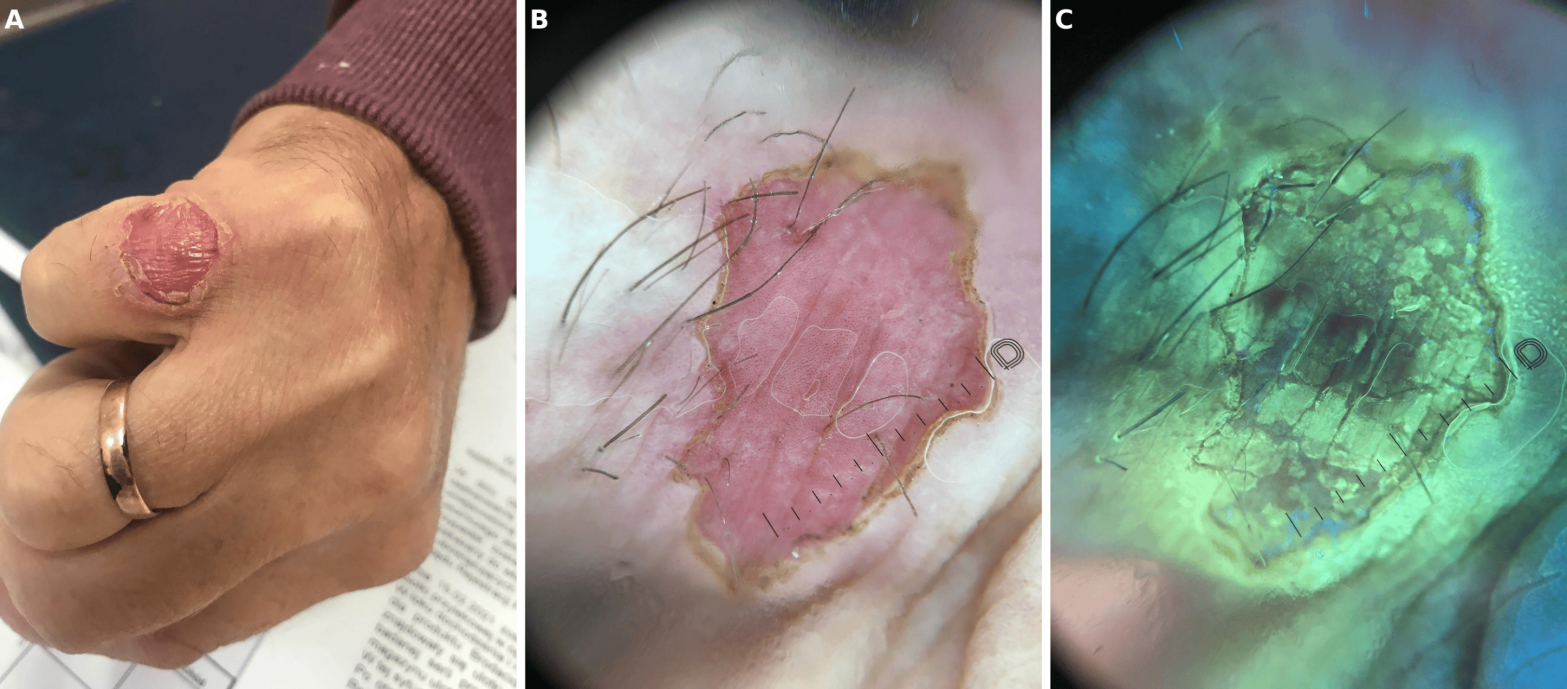
(a) Clinical photograph of a circular erosion located on the knuckle. The lesion has a well-demarcated border and features central scaling and erythema. (b) Dermoscopic image of the same lesion. Dermatoscopy revealed a double rim corresponding to cornoid lamella, dotted vessels, and a pink background. (c) Ultraviolet-induced fluorescence dermatoscopy reveals a yellow fluorescence due to some antiseptic specifics the patient used topically prior to his visit

A 56-year-old man presented to the dermatology clinic with an erosion at the base of the dorsal part of the fifth finger on his right hand. This lesion appeared two years earlier, following the application of a lactic and salicylic acid solution, which, as the patient reported, he used on a viral wart in the same location (Figure 1A). The patient states that it was not healing; however, we suspect it was most likely a primary cell lesion for PM. Additionally, the patient reports chronic diseases, including well-controlled diabetes, hypertension, and coronary artery disease previously treated with coronary angioplasty with stent implantation. His ongoing medications include metformin, empagliflozin, dulaglutide, perindopril, acetylsalicylic acid, and an HMG-CoA reductase inhibitor.

He trimmed the edges of the lesion and frequently cleaned a marine aquarium. He was consulted at a clinic for chronic wounds with no improvement after the treatment. A mycological examination of a sample from the ulcer was performed, and the result was negative.

Previously, his therapeutic modalities included creams, gels, and patches with silver ions, paraffin dressings, 15% urea cream, emollients and ointments containing flumetasone and salicylic acid, betamethasone, clotrimazole, and gentamicin, as well as tacrolimus.

Dermatoscopy revealed a double rim corresponding to cornoid lamella, dotted vessels, and a pink background (Figure 1B). Ultraviolet-induced fluorescence dermatoscopy reveals a yellow fluorescence due to some antiseptic specifics the patient used topically prior to his visit (Figure 1C).

**Figure 2:**
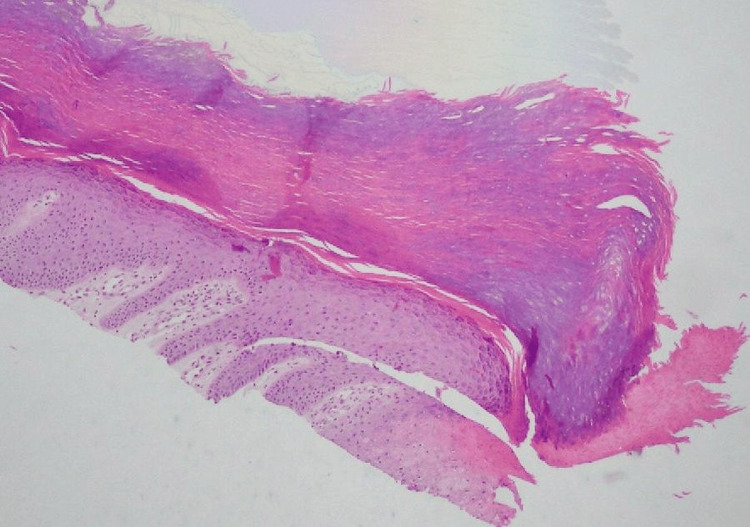
Parakeratotic column indented into the thick palmar epidermis with loss of the granular layer at its base

The patient was advised to use silver foam dressings to stop cleaning the mentioned saltwater aquarium himself and was scheduled for a follow-up visit for a further dermatoscopic evaluation of the lesion and moles. A biopsy of the skin lesion was performed, and the histopathological examination revealed a parakeratotic column indenting into the epidermis, consistent with porokeratosis. Based on the clinical picture and histopathology exam, PM was diagnosed (Figure 2).

**Figure 3:**
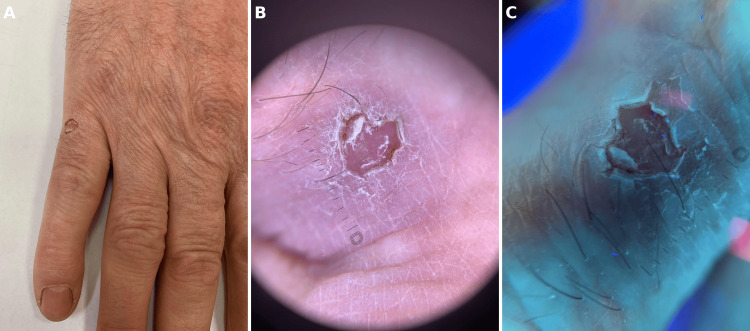
(a) Significant improvement after lovastatin/cholesterol therapy. The lesion is much smaller, with a crusty and scaly appearance. (b) Dermoscopic image presents double rim cornoid lamella and dotted vessels with a pink background. (c) Ultraviolet-induced fluorescence dermatoscopy reveals a white, double rim cornoid lamella

He started treatment with a topical 2% lovastatin/2% cholesterol ointment. After nine months of the aforementioned treatment, he returned to the clinic for a follow-up, showing marked improvement (Figure 3A). Dermatoscopy revealed double rim cornoid lamella and dotted vessels with a pink background (Figure 3B). Ultraviolet-induced fluorescence dermatoscopy reveals a white, double rim cornoid lamella (Figure 3C).

## Discussion

PM is a rare, chronic skin condition that often presents as either a single, outwardly expanding solitary plaque or multiple papules/macules. The lesions, characterized by central atrophy and raised keratotic borders, can vary significantly in size, reaching up to 20 cm in diameter. While these lesions may occasionally regress on their own, they are most commonly found on the limbs and can also manifest on other body parts, including the palms, soles, face, scalp, mucous membranes, and genitals [1].

Both familial cases with an autosomal dominant inheritance pattern and sporadic occurrences have been documented [3]. Additionally, porokeratosis is linked to an increased risk of developing skin cancer [4]. The aforementioned cornoid lamella in porokeratosis results from the expanding clonal proliferation of atypical keratinocytes, attributed to a genetic mutation [1]. Research has highlighted the significant role of four genes (MVK, PMVK, MVD, and FDPS) in the mevalonate pathway, which is crucial for the synthesis of cholesterol and other sterols, influencing cell growth and apoptosis [5]. Notably, the PM subtype of porokeratosis shows particular alterations in the MVK and PMVK genes, resulting in decreased expression of the respective enzymes [6,7].

Subsequently, mutations in the aforementioned genes result in a cholesterol deficiency. Cholesterol, a critical product of this pathway, is essential for maintaining skin barrier function. Deficiencies in cholesterol due to these genetic mutations may lead to increased apoptosis and altered keratinocyte behavior, which contribute to the development of porokeratosis [1]. Advances in treatment, particularly the topical application of lovastatin combined with cholesterol, aim to correct these metabolic disruptions by replenishing cholesterol levels and reducing toxic metabolite accumulation [8]. Emerging evidence supports the efficacy of cholesterol/statin therapy across various types of porokeratosis [9].

On the other hand, the recent finding suggests that the accumulation of mevalonate, rather than disruptions in cholesterol synthesis, is the more likely cause of porokeratosis [10]. This implies that targeting and blocking the mevalonate synthesis pathway upstream of the MVD enzyme could effectively alleviate symptoms by preventing the buildup of mevalonate pathway metabolites. Such findings suggest a limited benefit from adding cholesterol to the treatment. Regardless, the effectiveness of lovastatin monotherapy and combined cholesterol/statin therapy for various subvariants of porokeratosis requires further investigation.

## Conclusions

Studies show the efficacy of topical lovastatin/cholesterol use, especially in cases of LP and DSAP. This study underscores the effectiveness of a topical 2% lovastatin/2% cholesterol ointment in treating PM, presenting the first documented instance of significant clinical improvement for this subtype. Over nine months, the treated lesion demonstrated a marked reduction in size and enhanced appearance. This finding confirms the therapeutic potential of combining lovastatin with cholesterol in addressing the metabolic disruptions associated with PM and prompts a reevaluation of current therapeutic approaches, particularly in light of the persistent challenges in treating this complex condition.
